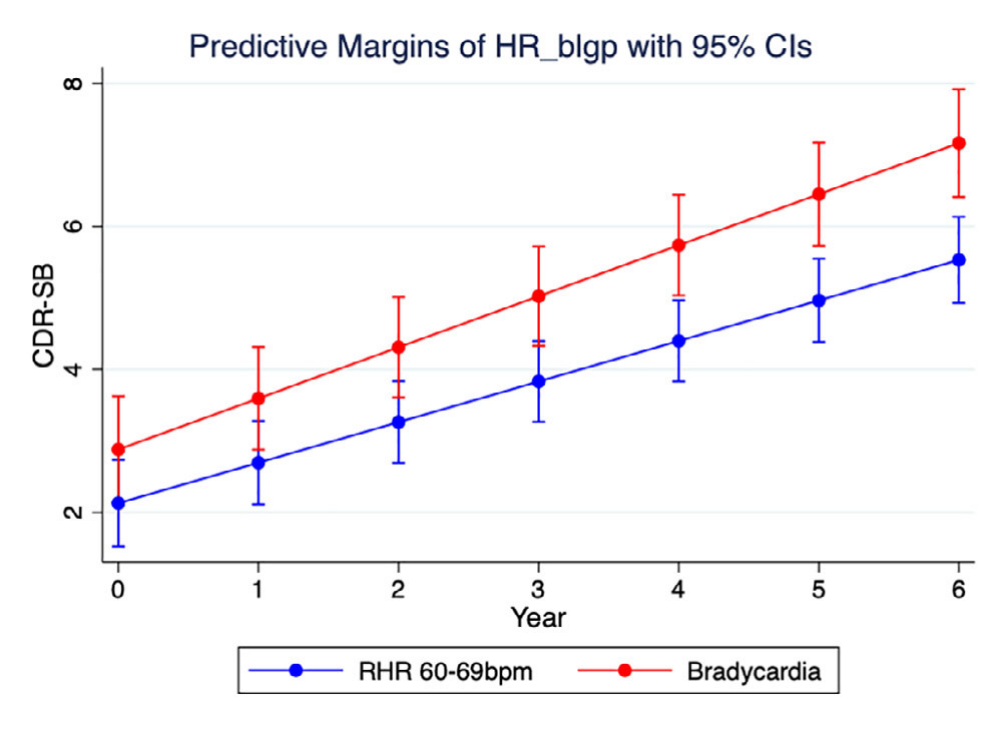# Association of bradycardia with longitudinal cognitive decline

**DOI:** 10.1002/alz70856_097314

**Published:** 2025-12-24

**Authors:** Lucia Li, Ming Ann Sim, Eugene Tan, Christopher Chen

**Affiliations:** ^1^ University of Cambridge, Cambridge, Cambridgeshire, United Kingdom; ^2^ National University of Singapore, Singapore, Singapore; ^3^ National University of Singapore, Singapore, Singapore, Singapore; ^4^ National University Hospital, Singapore, Singapore, Singapore; ^5^ National University Heart Centre Singapore, Singapore, Singapore, Singapore; ^6^ National University Health System, NUHS, Singapore, Singapore, Singapore; ^7^ Yong Loo Lin School of Medicine, National University of Singapore, Singapore, Singapore

## Abstract

**Background:**

Relationships between bradycardia and cognitive trajectories and the underlying mechanisms remain unclear. The present study investigated associations bradycardia with longitudinal cognitive decline, circulating biomarkers, and cerebrovascular disease among subjects without atrial fibrillation (AF).

**Method:**

Resting heart rate (RHR) was recorded in memory clinic subjects with comprehensive neuropsychological assessment, brain magnetic resonance imaging, and blood samples. Longitudinal associations between bradycardia (RHR <60 bpm), compared to 60–69bpm, and cognitive decline (up to 5 years), graded at baseline and annually with the Clinical Dementia Rating (CDR)‐sum of boxes score (CDR‐SB) were ascertained. Cross‐sectional associations between RHR and neuroimaging markers and circulating biomarkers were analysed.

**Result:**

Among 390 subjects without AF (mean age 73.0±7.9 years, 57% female, 156 (40%) were bradycardic. There were no differences in total education years, prevalence of hypertension, diabetes, hyperlipidemia, smoking, cognitive impairment without dementia (43% vs 38%) and dementia (36% vs 41%) between non‐bradycardia and bradycardic subjects (*p* >0.05). Compared to RHR 60‐69bpm, bradycardia was associated with accelerated cognitive decline (CDR‐SB) after adjustment for age, sex, education, apolipoprotein E4 status, hypertension, diabetes, hyperlipidemia, rate‐limiting medications and baseline cognitive impairment (ß 0.15, 95%CI 0.03‐0.26, p_interaction_*time=0.013) in linear mixed effects regression. Cross‐sectionally, bradycardia was associated with higher levels of plasma neurofilament light chain (*p* = 0.033) and phosphorylated tau‐181 (*p* = 0.003) levels, lower gray matter volume (*p* = 0.016), and higher counts of cortical infarcts (*p* = 0.049) compared to RHR 60‐60bpm.

**Conclusion:**

Bradycardia was associated with accelerated cognitive decline. Further study into the mechanisms underpinning this association is required.